# Examining visual fields

**Published:** 2019-12-17

**Authors:** David C Broadway, Fatima Kyari

**Affiliations:** 1 Consultant Ophthalmic Surgeon: Department of Ophthalmology, Norfolk & Norwich University Hospital, and Honorary Reader: University of East Anglia, Norwich, UK.; 2 Consultant Ophthalmologist Coordinator, College of Health Sciences Baze University Abuja, Nigeria.

**Basic visual field testing only takes a few minutes, but can help to detect glaucoma and macular disease.**

**Figure 1:**
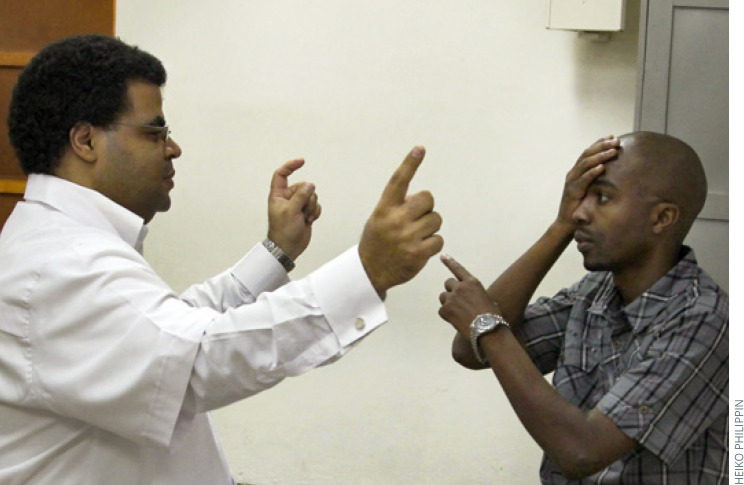
Testing visual fields to confrontation. The examiner's left eye is closed, so he can compare the field of his right eye with the field of the patient's left eye.

Examining visual fields is important for the detection of glaucoma, macular disease and neurological conditions such as stroke, and is an integral part of a full ophthalmic evaluation. In this article, we describe how to detect visual field defects using confrontation visual field testing and an Amsler Grid, neither of which requires expensive equipment.

Early (or even moderate) visual field defects often go unnoticed, particularly if only one eye is affected. The images in Figure 2 represent what a scene may look like to someone with different visual field defects in each eye. The left eye has inferior field loss, and the right eye has superior field loss. Because the defects do not overlap, the field defects will not be apparent when the scene is viewed with both eyes together.

Useful questions to ask are:

- Have you noticed if any part of your vision is missing in either eye?
- Have you noticed any gaps in your vision?
- If you close each eye in turn, does what you see differ from one eye to the other?

In addition, it is essential to enquire about past ophthalmic and medical history, concentrating on family history and whether there are any additional ophthalmic or neurological symptoms.

## Confrontation visual field testing

Confrontation visual field testing only takes a few minutes and can provide useful information. Prepare by testing yourself first so that you can become familiar with the range and limitations of your own field of vision and locate your blind spot in each eye. A defect is detected when you show a target and the patient does not react, even though it is at the same distance from you and the patient. The assumption is that you, as the examiner, have normal visual fields. This is another reason why you should undergo visual field testing yourself first.

During the examination, first test the binocular visual field (with both eyes open) and then test each eye separately. You will need a target: this can be a finger that waves, or curls and uncurls, or a pen with a red top.

### Confrontation testing with both eyes

Ask the patient to stare directly and steadily into your eyes. Staring can cause embarrassment or awkwardness, so allow the patient to rest and try again if they find it difficult to look at you directly. Check that the patient can look steadily at your eyes while you look steadily at theirs. Ask the patient whether any part of your face is missing or indistinct.

**Figure 2:**
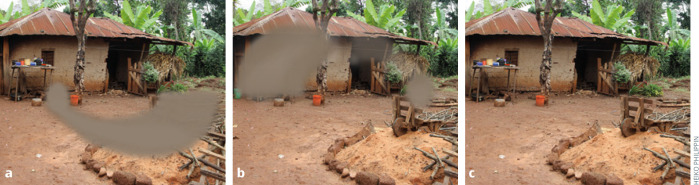
The left eye has inferior field loss (a), and the right eye has superior field loss (b). Because the defects do not overlap, they will not be apparent when the scene is viewed with both eyes together (c).

1. Check the patient's left hemi-field by making a fist with your right hand and holding it in their left hemi-field, at eye level, just to the right of your face. Making sure that the patient is still holding your gaze, raise one to four fingers and ask how many fingers can be seen. To test the upper and lower quadrants, move your hand up and to the right, and down and to the right, repeating the test at various points. This simple finger-counting test is particularly useful for detecting visual field loss due to neurological problems (such as strokes), but is only useful for patients with glaucoma when the visual field loss is severe.
2. To test the patient's right hemi-field and upper and lower quadrants, repeat the finger-counting test using your left hand, starting just to the left of your face and moving up and left and then down and left.

### Testing each eye to confrontation

1. Ask the patient to cover their own eye with the palm of their hand (not their fingers, as it is easy to peep between fingers). Remember that you should close your eyes in turn too, so that you are comparing the field in your right eye with the field of the patient's left eye, for example (Figure 1).
2. Do the finger counting test first (static testing). Be sure to test on both the left and the right for **each eye tested**.
3. Next, bring your target finger from the far periphery in towards the central region (kinetic testing). Ask the patient to say when they first see the target. Repeat from several different directions, ensuring that the full 360° for each eye is tested. The examiner should remember to perform kinetic testing at a speed appropriate for the patient's responses.
4. Next, test the peripheral (outer) field preferably with a white target (this can be a pin or eye drop bottle lid) and then test the central (inner) field with a red target (eye drop bottle lid or the top of a pen. Testing with these targets gives more accurate results than testing with fingers and can detect earlier visual field loss. In addition, red-headed targets can be used to test for red-desaturation. A sign of early optic nerve disease.

## Amsler chart testing

**Figure 3:**
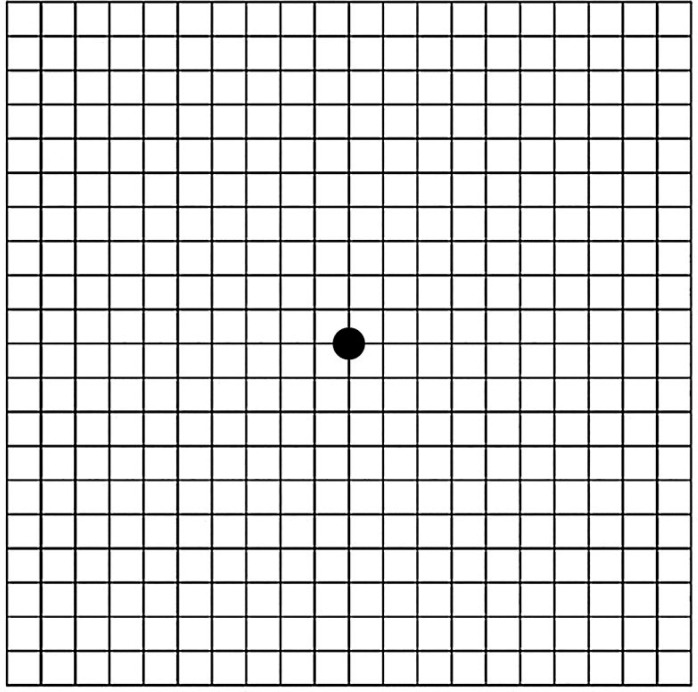
Amsler grid, used to check distortion of central vision

A printed grid, known as an Amsler grid (Figure 3) can be used to detect abnormalities in the central field as well as paracentral defects (fairly common in patients with glaucoma).

Test one eye at a time, correcting for any near refractive errors. Ask patients to hold the chart at a comfortable reading distance from their uncovered eye, and stare at the central spot of the grid. Ask them to identify and then point to any areas where the grid is missing or distorted. Missing areas may suggest paracentral glaucomatous visual field loss, whereas distortion is more common with macular disorders.

**Figure 4:**
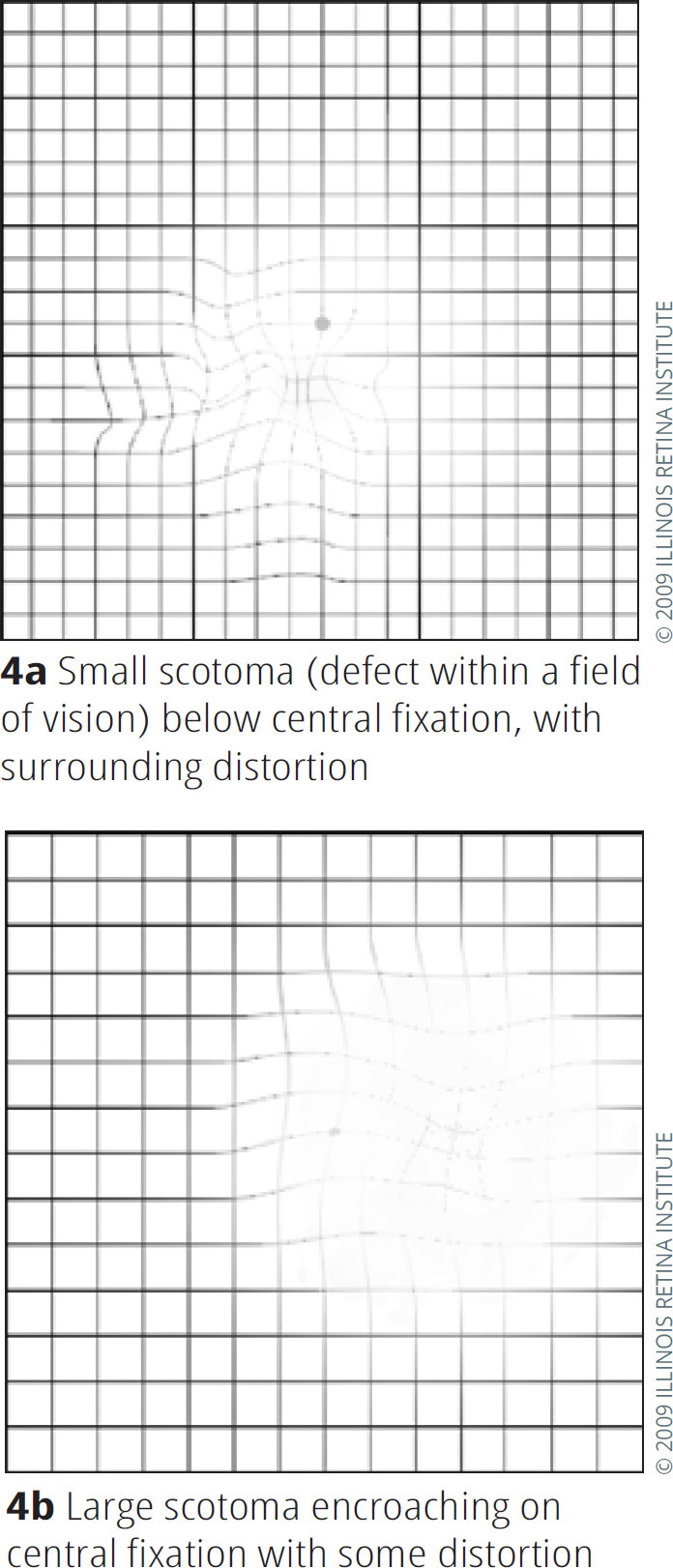
Amsler grid when viewed by someone with a problem with their central visual field (a and b)

